# A Dynamic Scheduling Method of Earth-Observing Satellites by Employing Rolling Horizon Strategy

**DOI:** 10.1155/2013/304047

**Published:** 2013-04-18

**Authors:** Qiu Dishan, He Chuan, Liu Jin, Ma Manhao

**Affiliations:** Science and Technology on Information Systems Engineering Laboratory, National University of Defense Technology, Changsha 410073, China

## Abstract

Focused on the dynamic scheduling problem for earth-observing satellites (EOS), an integer programming model is constructed after analyzing the main constraints. The rolling horizon (RH) strategy is proposed according to the independent arriving time and deadline of the imaging tasks. This strategy is designed with a mixed triggering mode composed of periodical triggering and event triggering, and the scheduling horizon is decomposed into a series of static scheduling intervals. By optimizing the scheduling schemes in each interval, the dynamic scheduling of EOS is realized. We also propose three dynamic scheduling algorithms by the combination of the RH strategy and various heuristic algorithms. Finally, the scheduling results of different algorithms are compared and the presented methods in this paper are demonstrated to be efficient by extensive experiments.

## 1. Introduction

The mission of an earth-observing satellite (EOS) is to scout targets with a certain range of ground to produce high-resolution photographs [1–4]. According to the working mechanism, the imaging sensors load in EOS can be divided into visible light imaging, microwave imaging, infrared imaging, and so forth. Since they have numerous merits such as rapider response, broad coverage range, longer duration, and freedom from airspace boundaries, EOS supports many important services, such as military surveillance, geodesy and navigation, remote sensing, and monitoring.

Nowadays, EOS is attracting more and more interests worldwide be accompanied with the dramatic increase of the demand for imaging service. One major research trend is that the single satellite used in early reconnaissance is replaced by cooperation of large satellites, yielding the socalled multi-satellite application. Unfortunately, as the number of satellites grows large, the traditional manual coordination will no longer be feasible because multisatellite scheduling (MSS) is an NP-hard combinatorial optimization problem [5]. Therefore, the researches on MSS refer to assigning observation resource to match various imaging requirements as indispensable. 

There exist numerous studies on scheduling algorithm for multi-satellite to realize automated resource planning. Wang et al. [6] proposed a hybrid ant colony optimization (HACO) algorithm to overcome the disadvantage of current ant colony optimization (ACO) algorithm which is more easily plunged into local optimal solution in solving MSS problem. Jian and Cheng [7] constructed an integer programming model of MSS based on the analysis of the resource constraints and task characteristic. To solve this combinatorial optimization problem, they provided a genetic particle swarm optimization (PSO) algorithm which searches only in the appointed integer space. He et al. [8] presented a cooperative scheduling architecture of multiple satellites by converting this scheduling problem into a main problem and a subproblem. In addition, an improved PSO algorithm was used to solve MSS problem by taking task benefits as an optimization objective. 

 However, those above researches have been primarily focused on static scheduling problem of EOSs. It is usually assumed that the imaging tasks have been submitted before scheduling and their information is acquired. In practice, the requests from customers are continuously delivered, which lead to the imaging tasks arriving one by one. The most significant feature of dynamic scheduling is time urgency; that is, the task must be completed within a specified time limit, or it will lose its execution value caused by failure. The satellite imaging reconnaissance mission generally has a deadline, which can reflect its execution urgency. Execution of the task must be completed within the specified deadline; otherwise, the expected benefits will not be obtained. The traditional static scheduling methods tend to overlook the timeliness feature of imaging task, which makes them inapplicable to the dynamic scheduling problem of imaging satellite.

At present, there are a few works on the dynamic scheduling problem of EOSs. Baolin et al. [9] described a new satellite mission scheduling algorithm based on constraint satisfaction problem (CSP). Zhu et al. [10] considered the problems of satellite scheduling for realizing optimal disaster rescue and proposed a hybrid algorithm to solve this multiple objects optimization problem. Wang et al. [11] considered the characteristic of EOS in dynamic scheduling and proposed a rule-based heuristic algorithm to solve this problem. The study mentioned above still has the following shortages.

1. It is difficult for these proposed algorithms to generate a task planning within a short time. A dynamic planning process consists of repeated scheduling events, and the traditional intelligence algorithm (IA) has high timing complexity, which cannot rapidly generate the planning scheme. Therefore, the high-efficiency heuristic algorithm should be used to address the dynamic scheduling problem of EOSs.
2. The impacts of scheduling time on the available tasks were not considered. Since tasks are dynamic arrivals, the planning system collects dissimilar task sets at different scheduling times. Thus, the task set should be determined based on current scheduling time before the scheduling.
3. The constraints during dynamic scheduling have not been adequately considered. Many constraints (i.e., the storage capacity, maximum swing angle, and continuous observation time) which have been simplified in static scheduling should be considered in dynamic scheduling.

In this paper, we tackle the above challenges imposed on the dynamic scheduling problem of EOSs by handling the impacts of deadline constraint and scheduling time on planning scheme. The integer programming model is constructed based on various constraints in actual reconnaissance activities, and the rolling horizon (RH) strategy and heuristic algorithms are employed to solve this model.

The remainder of this paper is organized as follows.  Section 2 describes the dynamic scheduling problem of EOSs, and establishes the integer programming model.  Section 3 designs the scheduling architecture and algorithms based on the RH optimization. Simulation results and performance analysis are given in Section 4. Finally, Section 5 concludes the paper with some future research directions.

## 2. Problem Description and Modeling

**Figure 1:**
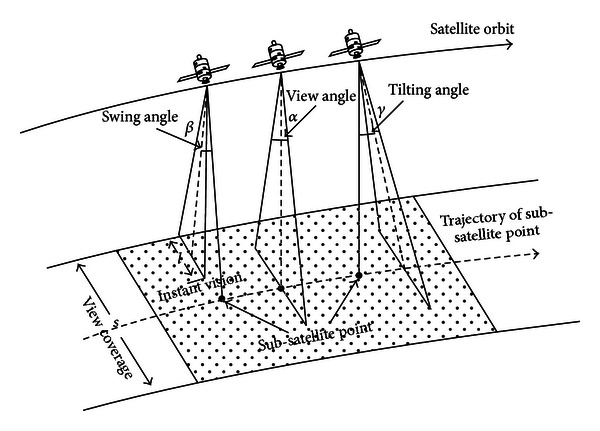
The observation field of EOS.

The EOS operates in the space in a certain orbit as shown in Figure 1. The view coverage of EOS can be formed on the ground by the subsatellite point of satellite platform as well as the view angle, swing angle, and tilting angle of satellite payload [12–14].

The purpose in addressing the dynamic scheduling problem of EOSs is to appoint observation resources and execution time for the dynamical submitted tasks with various constraints, so as to maximize the task benefits of reconnaissance activity and minimize the resource consumption as far as possible.

The imaging tasks in dynamic scheduling problem are submitted to the planning system in independent times compared to the static scheduling problem which can obtain all the tasks in advance. The dynamic scheduling system only acquires the information of arrived tasks but can not gain the situation of following tasks. Hence it needs to trigger multiple scheduling in order to cope with the new tasks which are successively submitted to the scheduling system. Therefore, the dynamic scheduling algorithms designed in this paper should have the overall coordination capacity; that is, the algorithm should be able to timely adjust the execution scheme of planned tasks for executing the emergency tasks submitted later, so as to maximize task benefits. For future reference, we summarize main notations used in this paper as the following:

  
*T*_*p*_ = [*t*_start_, *t*_end_] is the reconnaissance activity period of satellites, where *t*_start_ is the starting time and *t*_end_ is the ending time of the observation activity;
  
*T*_S_ = {*t*_*S*_^0^, *t*_*S*_^1^,…, *t*_*S*_^*p*^} is the scheduling time set, where *t*_*S*_^*i*^  (*i* ≥ 1) is the *i*th scheduling time, *t*_*R*_^0^ = *t*_start_ is the virtual initial scheduling time, *T*_period_ = (*t*_*S*_^*p*^ − *t*_*S*_^0^)/*p* is the average scheduling period, and *p* is the quantity of scheduling activities during the reconnaissance activity period;
  Task = {task_1_, task_2_,…, task_*m*_} is the imaging task set, where task_*i*_ is the *i*th task, and *m* is the task number;
  Sat = {sat⁡_1_, sat⁡_2_,…, sat⁡_*n*_} is the satellite resource set, where sat⁡_*j*_ is the *j*th satellite, and *n* is the satellite number;
  
*TW*_*i*_ = ⋃_*j*=1_^*n*^⋃_*k*=1_^*q*_*i*,*j*_^ *tw*_*i*,*j*_^*k*^ is the observation opportunity window set of task_*i*_ between its arrival time *a*_*i*_ and deadline *d*_*i*_, where *q*_*i*,*j*_ is the opportunity window number of task_*i*_ on sat⁡_*j*_, *tw*_*i*,*j*_^*k*^ = [*ws*_*i*,*j*_^*k*^, *we*_*i*,*j*_^*k*^] is the *k*th opportunity window of task_*i*_ on sat⁡_*j*_, and *ws*_*i*,*j*_^*k*^ and *we*_*i*,*j*_^*k*^ are the starting time and ending time of *tw*_*i*,*j*_^*k*^, respectively.

In addition, task_*i*_ has an execution benefit *p*_*i*_ and it requires a continuous observation time *d*_*i*_. If task_*i*_ can be executed before its deadline, assume *ts*_*i*_ is the starting time, *te*_*i*_ is the ending time, and *h*_*i*_ is the required storage capacity. Furthermore, the satellite has a swing angle *gh*_*i*_ and a tilting angle *gw*_*i*_ to execute task_*i*_. Assume *a*_*j*_ is the view angle of sat⁡_*j*_, *β*_*j*_ is the maximum swing angle, *γ*_*j*_ is the maximum tilting angle, *ω*_*j*_ is the swing speed, *ρ*_*j*_ is the tilting speed, *tc*_*j*_ is the position transfer stabilization time, *r*_*j*_ is the maximum position transfer frequency, and *M*_*j*_ is the memory capacity.

The decision of scheduling times is affected by many factors, for example, the quantity and density of task, the upload period of satellite instructions, and the communication capacity of control center. The decision variable of the dynamic scheduling problem provided in this paper is as follows:

(1) $$\begin{array}{c} x_{i,j}^{k}=\{\begin{array}{cc} 1, & \text{if}\;{\text{task}}_{i}\;\text{is}\;\text{executed}\;\text{in}\;tw_{i,j}^{k},\\ 0, & \text{otherwise}. \end{array} \end{array}$$

Assume *t*_*S*_^*k*^ is the current scheduling time and the maximum task benefits are the optimization object, then we can build the integer programming model of the dynamic scheduling problem as follows [15]:

(2) max⁡ Z=∑i=1m∑j=1n∑k=1qi,jxi,jkpis.t. {∑jn∑kqi,jxi,jk≤1, [tsi,tei]⋂[tsi′,tei′]=∅,      (1)ai≤tSk≤tsi, if  ∑j=1n∑k=1qi,jxi,jk=1,           (2)[tsi,tei]⊂⋃k=1qi,jtwi,jk⊂[tSk,ei], if  ∑k=1qi,jxi,jk=1,          (3)tei−tsi≥di, if  ∑j=1n∑k=1qi,jxi,jk=1,         (4)tei+max⁡(|ghi−ghi′|ωj,|gwi−gwi′|ρj)+tcj≤tsi′, if  (tei≤tsi′)∧(xi,jk=xi′,jk′),    (5)∑im∑kqi,jxi,jkhi≤Mj,          (6)∑im∑kqi,jxi,jk≤rj,           (7)   i,i′∈[1,2,…,m],j∈[1,2,…,n]k∈[1,2,…,qi,j], k′∈[1,2,…,qi′,j],

where *Z* is the optimization objective; constraint (1) means each task only need to be executed once, and the execution process does not involve preemptive service; constraint (2) means the scheduling system only assigns the arrived tasks and their deadline must later than current scheduling time; constraint (3) means that if any task is executed, the execution time should between current scheduling time and its deadline; constraint (4) means that if the task can be executed, its execution time should not be shorter than the required continuous observation time; constraint (5) means any two tasks assigned to the same satellite should have an adequate time interval between their execution times to ensure the sensor of satellite can adjust its gesture; constraint (6) means that the memory store any task should not exceed its maximum capacity; constraint (7) means that the gesture transfer number of satellite in one orbit should not be higher than the allowable frequency.

## 3. Dynamic Scheduling Approach

### 3.1. Architecture of Algorithm

In this paper, the dynamic scheduling algorithm based on the RH strategy [16–18] is proposed to allocate tasks which have special characteristic such as the independent arrival time and deadline. This method can conduct scheduling (or rescheduling) forward for a task set through rolling advance, so as to effectively handle the uncertain factors brought by the dynamic tasks. The heuristic algorithms are incorporated with RH strategy to reduce the timing complexity of scheduling while each scheduling requires one replanning. In other words, the task set that needs to be scheduled is determined by the RH strategy firstly, and then the heuristic algorithms are used to assign satellites and execution periods to each task.

### 3.2. Rolling Horizon Strategy

The basic method of RH strategy is to divide the tasks into multiple task sets with certain overlaps based on the arrival sequence, and the division can be continuously updated along with the scheduling time. Each scheduling will decide and only assign its task set, which is called as rolling horizon. The new tasks are continuously added to the rolling horizon, and the finished tasks are gradually deleted with the advancement of the scheduling time, so as to realize the update of rolling horizon. The advantage of RH strategy is that it can decompose the complicated dynamic scheduling problem into multiple simple static scheduling sub-problems, and the optimization solution of previous problem is replaced with the optimized solutions of sub-problems, so that the complexity of the original problem will be reduced.

#### 3.2.1. Task States

In general, tasks will go through four states based on current scheduling time: a new task, waiting task, running task, and finished task. One task may be scheduled in different time, thus the state of task is dynamic; that is, a task might be in two states in different scheduling.

**Figure 2:**
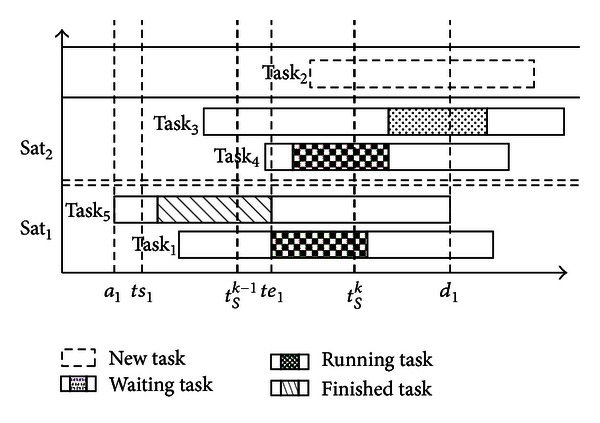
Task states based on current scheduling time.

In the example shown in Figure 2, *t*_*S*_^*k*^ is current scheduling time. If task_*i*_ has been planned during the (*k* − 1)th scheduling (i.e., *a*_*i*_ ≤ *t*_*S*_^*k*−1^), then task_*i*_ is a finished task while *te*_*i*_ ≤ *t*_*S*_^*k*^ (such as task_1_); task_*i*_ is a running task if *tb*_*i*_ < *t*_*S*_^*k*^ ≤ *te*_*i*_; task_*i*_ is a waiting task if *tb*_*i*_ > *t*_*S*_^*k*^. If task_*i*_ has not been planned in the (*k* − 1)th scheduling (i.e., *t*_*S*_^*k*−1^ ≤ *a*_*i*_ ≤ *t*_*S*_^*k*^), then task_*i*_ is a new task.

#### 3.2.2. Rolling Horizon

The rolling horizon is used to store the tasks that need be scheduled currently. There are two key elements about rolling-horizon: the quantity and state of tasks in rolling-horizon. From the perspective of task quantity, the more the tasks fall into the rolling horizon, the stronger the capacity of scheduling system to obtain comprehensive task information is, which is important to acquire the better solution. But the timing complexity of scheduling algorithm will also be aggravated. From the perspective of task state, the rolling-horizon consists of the running tasks, waiting tasks, and new tasks generally. Among them, the processing method for the running task is an important criterion to distinguish the preemptive and nonpreemptive scheduling. The later scheduling mode is out of the interest of this paper; that is, the rolling-horizon only includes the waiting tasks and new tasks. It should be noticed that during actual scheduling, the rescheduling of waiting task will not consume any additional resources because waiting task has not been executed yet.

#### 3.2.3. Trigger Mode

The arrangement of scheduling time is the key factor which affects the application efficiency of RH strategy, and it is mainly determined by the trigger mode of scheduling. The general trigger modes include the following types.

 (i) *Event Trigger Mode. *The scheduling is triggered while the scheduling environment is changed or a manual intervention occurred, such as a new task arrived, satellite number changed, or a scheduling requirement was sent from decision-making section. The event-trigger mode is sensitive to the scheduling environment, and it can assign the emergency tasks in time. However, this mode might cause the scheduling algorithm to have a high timing complexity due to frequent scheduling, and result in hard to generate a planning scheme rapidly.

 (ii) *Period Trigger Mode.* In this mode, the scheduling event will be trigged after a certain time interval, and this time interval can be a uniform constant or dynamic variable. The period-trigger mode has capability to ensure the stable frequency of scheduling, but it can not provide timely scheme for emergency tasks with high timeliness, and also has the fault that it can not adjust scheme in dealing with the change of the satellite number.

 (iii) *Mixed Trigger Mode. *Mixed-trigger mode is the combination of the aforementioned two modes. It can timely allocate the emergency tasks with less time consumption, so it is an adaptive mode to be employed in the scheduling of real-time system and dynamic system.

The mixed-trigger mode is adopted in this paper, and the scheduling time for period factors and event factors is embodied in the elements belonging to *T*_*s*_. The RH strategy based on the mixed-trigger mode is depicted as follows.

In Algorithm 1, each scheduling time *t*_*S*_^*k*^ ∈ *T*_*S*_ will trigger one rescheduling (see line 1), and the scheduling frequency during *T*_*p*_ is *O*(*P*). The criterion described in Figure 1 is used to divide the arrived tasks into the finished task, running tasks, waiting tasks, and new tasks based on current scheduling time (see lines 3~12); the timing complexity is *O*(*m*). The tasks in rolling-horizon are sorted and assigned to the satellite resources and execution time (see line 13). The timing complexity of this step is mainly depends on the assigning algorithm; thus we assume it is *O*(*A*) temporarily. It only takes *O*(1) to execute other lines. Hence, the timing complexity of Algorithm 1 is calculated as *O*(*P*)[*O*(*m*) + *O*(*A*)].

The above algorithm needs to assign satellite resources and execution time for each task, so *O*(*A*) is usually much larger than *O*(*m*). The timing complexity of Algorithm 1 will be dominated by *O*(*A*) if the scheduling frequency (or scheduling interval) is a constant. Then, the additional timing complexity caused by RH strategy is mainly related to the scheduling frequency but not affected by other factors such as the satellite quantity. At present, the intelligence algorithms (IA) are usually used to assign satellites and execution time to tasks in the static scheduling problem. These algorithms generally require multiple iterations to realize optimization of the scheduling scheme, and have a high timing complexity. The imaging tasks in dynamic scheduling have the timeliness feature, which requires the planning scheme to satisfy the deadline possibly, and, furthermore, the planning scheme should be rapidly generated and can be fast adjusted to adapt to the change of task set by scheduling algorithm. In Algorithm 2, the heuristic algorithm is employed to match the satellites and the tasks in order to shorten the generation time of the planning scheme. Meanwhile, the RH strategy can well adjust the previous planning scheme of planned tasks based on the change of rolling-horizon. The timing complexity of the heuristic algorithm embedded in RH strategy will be analyzed in detail in the next section.

### 3.3. Heuristic Algorithms

This paper has proposed the heuristic algorithms AIS, DIS, and WIS based on the arrival time priority, the deadline priority, and the waiting time priority inspired by the earlier arrived time first (EAT) algorithm [19] and earlier deadline first (EDF) algorithm [20, 21] and considering the task importance.

Let RH = {task_*k*1_, task_*k*2_,…, task_*km*_} denote the rolling-horizon in the *k*th scheduling. The basic parameters (including the arrival time, deadline, waiting time, and importance) of all tasks are standardized as follows:

(3) $$\begin{array}{c} f\left(x_{k_{i}}\right)=\frac{x_{k_{i}}-\min_{\text{tas}{\text{k}}_{k_{i}}\in \text{RH}}\left(x_{k_{i}}\right)+1}{\max_{\text{tas}{\text{k}}_{k_{i}}\in \text{RH}}\left(x_{k_{i}}\right)-\min_{\text{tas}{\text{k}}_{k_{i}}\in \text{RH}}\left(x_{k_{i}}\right)+1}. \end{array}$$

After the standardization, we record the basic parameters of task_*k*_*i*__ as AS_*k*_*i*__, DS_*k*_*i*__, CS_*k*_*i*__, and WS_*k*_*i*__ respectively. The arrival time priority degree of task_*k*_*i*__ is given as follows:

(4) $$\begin{array}{c} \text{A}{\text{I}}_{k_{i}}=\frac{\text{A}{\text{S}}_{k_{i}}}{\text{W}{\text{S}}_{k_{i}}}. \end{array}$$

The deadline priority degree of task_*k*_*i*__ is as follows:

(5) $$\begin{array}{c} \text{D}{\text{I}}_{k_{i}}=\frac{\text{D}{\text{S}}_{k_{i}}}{\text{W}{\text{S}}_{k_{i}}}. \end{array}$$

The waiting time priority degree of task_*k*_*i*__ is as follows:

(6) $$\begin{array}{c} \text{C}{\text{I}}_{k_{i}}=\frac{\text{C}{\text{S}}_{k_{i}}}{\text{W}{\text{S}}_{k_{i}}}. \end{array}$$

The main steps of the heuristic algorithm adopted in this paper are described as follows.

**Step 1**

The three priority degrees of each task in rolling-horizon are calculated.

**Step 2**

The tasks in the rolling-horizon are sorted by their different priority-degrees, and the ranking results can be obtained corresponding to the AIS, DIS, and WIS algorithms, respectively.

**Step 3**

The assignment strategy based on windows conflict index (WCI) is used to assign satellites and execution time for each task based on ranking results, and the scheduling scheme is generated after all tasks have been assigned.

In the aforementioned steps, WCI denotes the total impact on the unassigned tasks when a task is allocated to an available opportunity window. If task_*i*_ is appointed to the *k*th opportunity window of sat⁡_*j*_, the WCI of task_*i*_ can be calculated as follows:

(7) $$\begin{array}{c} \text{WC}{\text{I}}_{i,j}^{k}=\sum_{\text{tas}{\text{k}}_{i^{\prime }}\in \text{Waitin}{\text{g}}_{i}}\;\sum_{k^{\prime }=1}^{q_{i^{\prime },j}}\frac{g\left(tw_{i,j}^{k},tw_{i^{\prime },j}^{k^{\prime }}\right)}{we_{i^{\prime },j}^{k^{\prime }}-ws_{i^{\prime },j}^{k^{\prime }}}p_{i^{\prime }}, \end{array}$$

where Waiting_*i*_ is used to store tasks scheduled after task_*i*_ according to the ranking result and *g*(*tw*_*i*,*j*_^*k*^, *tw*_*i*′,*j*_^*k*′^) can be calculated as follows:

(8) g(twi,jk,twi′,jk′) ={0,if  wi,jk⋂twi′,jk′=∅,min⁡(wei,jk,wei′,jk′) −max⁡(wsi,jk,wsi′,jk′),otherwise.

Let Queue = {task_*b*1_, task_*b*2_,…, task_*bf*_} denote the task ranking result and *E*Queue the finished task set delivered by Algorithm 1. The pseudocode of heuristic algorithm is described in Algorithm 2.

In Algorithm 2, the tasks in the rolling horizon are scheduled in order (see line 1), and the timing complexity is *O*(*m*). The valid opportunity windows of task_*i*_ are checked to analyze the confliction with the running tasks (see lines 3~9); the timing complexity is *O*(*Tnm*). It takes *O*(*Tn*) to calculate the WCI of the valid opportunity windows (see lines 11~13). The timing complexity of other lines is *O*(1), so the timing complexity of Algorithm 2 is *O*(*m*)[*O*(*Tnm*) + *O*(*Tn*)] = *O*(*Tnm* ^2^). Furthermore, the timing complexity of Algorithm 1 is added to Algorithm 2; then the dynamic scheduling algorithm proposed in this paper has a timing complexity *O*(*P*)[*O*(*m*) + *O*(*Tnm* ^2^)] = *O*(*P* *Tnm* ^2^). Specially, the scheduling number and the reconnaissance activity period have a proportional relation if the rescheduling time interval is constant, then the timing complexity of dynamic scheduling algorithm is *O*(*T* ^2^ *m* ^2^ *n*).

We incorporate RH strategy with AIS, DIS, and WIS to yield three new algorithms named RH-AIS, RH-DIS, and RH-WIS, respectively. Meanwhile, AIS, DIS, and WIS can also be used to solve the dynamic scheduling problem separately; that is, only new tasks are scheduled in each scheduling by those heuristic algorithms. The six algorithms mentioned before are compared in the following experiment to evaluate the efficiency of the RH strategy.

## 4. Evaluation

The proposed algorithms are implemented by Matlab2007 on a laptop with Pentium IV 3.06 GHz CPU, 2 GB memory, and Windows XP operating system. The experimental scenarios are generated randomly for there has been no benchmark in the field of satellite scheduling yield. The operating points of simulated experiment are given as follows [22–24].

1. The reconnaissance activity period is from March 21, 2010, to March 22, 2010, and the scheduling period is two hours; that is, *T*_*R*_ = {0 h, 2 h,…, 24 h}.
2. The imaging tasks are generated in the area with a longitude 0°~150° and latitude −30°~60° randomly. The task quantity varied from 100 to 400. The arrival time gap between two adjacent tasks is subject to the negative exponential distribution, with a density of 0.1. Set the execution value of task from 1 to 10, the required continuous time 3~5 minute, and the occupied storage 2~4 G, and the deadline is a random variable generated between the arrival time of the task and the ending time of the observation activity, which abides by the uniform distribution.
3. The satellite quantity varied from 4 to 6, the memory storage is 240 G, the field angle is 3°, the maximum sway angle is 35°, the maximum tilting angle is 40°, and the maximum number of position transfers within a single orbit is no more than 5.

For the convenience of description, the dynamic scheduling problem of *n* satellites and *m* tasks is recorded as *m* × *n*. The twelve experiments are designed in different problem scales, and the performance metrics include the task benefits and guarantee ration. The scheduling results of various algorithms are displayed in Table 1.

From Table 1, we can observe that the RH strategy can effectively improve the performance of heuristic algorithms. The task benefits of the three heuristic algorithms embedding RH strategy increase by 16.19%, 15.82%, and 8.29%, respectively. This is because the algorithms without embedded RH strategy only operate the new tasks, while the embedded one can adjust the scheduling scheme of waiting tasks for executing the new tasks which can not be executed previously; hence a better scheduling solution can be achieved. Among the rolling-horizon scheduling algorithms (RH-AIS, RH-DIS, and RH-WIS), the task benefits obtained by RH-WIS are slightly higher than those of RH-DIS and RH-AIS while the satellites have adequate observation capacity to execute imaging tasks (for the problem scales 100 × 4, 100 × 5, and 100 × 6), and the resolution of RH-DIS is better than that of the other algorithms while the satellites have inadequate observation capacity. This is because both RH-DIS and RH-WIS have considered the deadline feature of tasks in scheduling scheme; it is the precondition to execute the valuable task. A large amount of high-timeliness tasks cannot be executed in their deadlines if the satellites have inadequate observation capacity, then RH-DIS gives priority to the tasks with earlier deadlines, so that the task guarantee ration is increased, and higher task benefits can be obtained. Most tasks can be effectively executed if the satellites have adequate observation capacity, then RH-AIS schedules tasks based on the deadline distribution but also considers the impact of executing current task on follow-up tasks, so that higher task benefits can be obtained on the aspect of overall planning effects.

**Figure 3:**
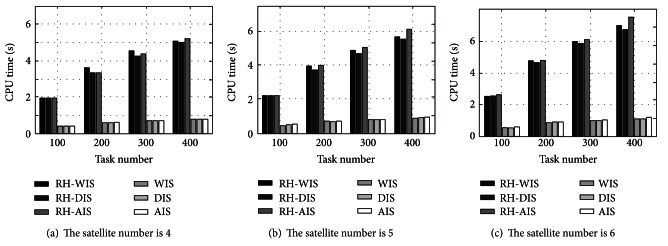
Search time of algorithm in different satellite quantities.

From Figure 3, the CPU time of six algorithms is compared in different satellite numbers. The timing complexity of scheduling algorithms is aggravated after embedding the RH strategy; thus the generation speed of the planning scheme is reduced. In the scenarios with three different satellite numbers, the average CPU times of algorithms incorporating RH strategy are increased 3.76, 3.73, and 3.74 times, respectively. The increased CPU time of rolling-horizon scheduling does not have special change with raising satellite number, which means the impact caused by RH strategy on the timing complexity of scheduling algorithm is weakly related to the satellite number, and this is consistent with the previous conclusion in the timing complexity analysis of Algorithm 2. Although the RH strategy will delay the generation of planning scheme, it also satisfies the timelessness of dynamic scheduling because the longest CPU time of the rolling-horizon scheduling is only 7.5 s, which is a low level.

**Figure 4:**
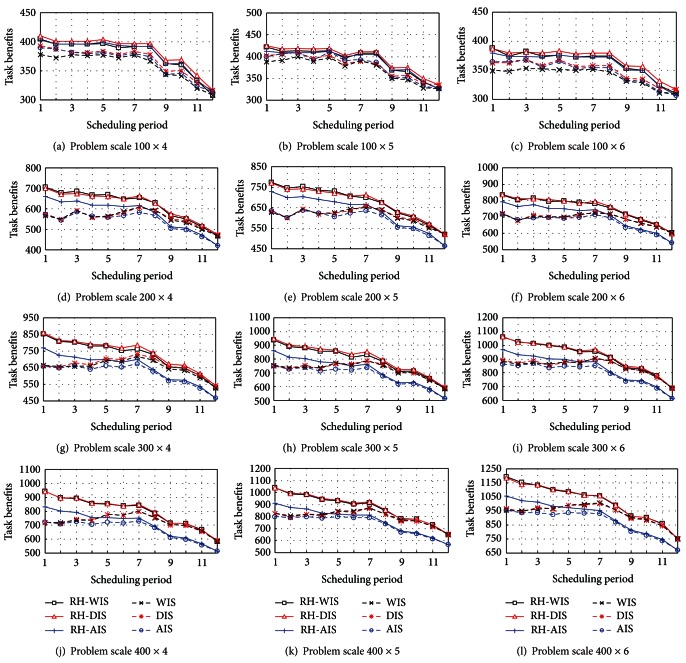
Performance of algorithms in different problem scales.

Set the scheduling period from one to twelve hours in order to analyze the impact of scheduling time interval on the overall performance of planning algorithm. The six algorithms are tested in different problem scales and shown in Figure 4.

From Figure 4, the task benefits are reduced overall as the scheduling period extends gradually. This is because the extensive scheduling period will increase the rejected tasks for their deadline can not be satisfied; hence the low task benefits are obtained for satellites unable to execute the tasks in time. The heuristic algorithms which have not been embedded into RH strategy (including RH-AIS, RH-DIS, and RH-WIS) still have high task benefits in different scheduling period. Let the ratio between the task number *n* and the satellite number *m* as the approximate workload of the satellite resources; then a scheduling period between 1~8 h does not have a significant impact on the task benefits if the satellite resource has a light workload. However, the task benefits will be sharply reduced if the scheduling period is extended to 8~12 h. There are high task benefits if we maintain the scheduling period within 1~6 h to adapt the increased workload of the satellite resource. It is also appropriate to maintain the scheduling period within 1~3 h in accordance with the heavier workload. Furthermore, it should be ensured the scheduling period is no more than 2 h while the satellite resource has the heaviest workload. So far we conclude that the scheduling time is a key factor to affect the task benefits. In addition, the task benefits do not present monotonic change in accompany with the scheduling period as shown in Figure 4. For example, Figure 4(a) presents that the task benefits gradually increase with the extension of scheduling period within 1~4 h and fluctuate within 4~8 h but begin to reduce within 8~12 h. This is because there is a conflict between the performance scheduling requirement of the planning system and the timeliness scheduling requirement of the tasks. The scheduling system only acquires the local task information if the scheduling period is short and yields inferior scheme which can not handle the following tasks with high timelessness. On the contrary, a large amount of high-timeliness tasks may not be finished before their deadline if the scheduling period is long, and the task benefits are reduced due to a low guarantee ration. Therefore, the scheduling period should be reasonably selected based on the satellite quantity and task density in actual application, so as to ensure the performance of scheduling system.

## 5. Conclusions and Future Work

This paper has studied the dynamic scheduling problem of EOSs. An integer programming model has been constructed by considering the independent arrival time and deadline of the imaging tasks. A dynamic scheduling algorithm based on the RH strategy which can be combined with multiple heuristic algorithms proposed in many researches and its timing complexity has been analyzed. 

The scheduling algorithms adapted to RH strategy can effectively adjust to the planning scheme based on the satellite workload to execute the emergency tasks. The effectiveness of this strategy has been verified by comparing the scheduling results of six algorithms in the experiment. It is worth to restate that the RH strategy might cause a high time consumption to yield an optimized scheme if a large amount of tasks are involved in the scheduling system. This problem can be solved by limiting the size of the rolling horizon. 

Also for our future work, we plan to research the detection method of scheduling time, which is a significant factor to impact the performance of scheduling algorithm and system. With the method in place, we will extend our algorithm to cooperative scheduling of EOSs; we will consider Qos requirements in our RH-WIS; we are going to combine the dynamic resources management into our scheme.

## Figures and Tables

**Algorithm 1:**
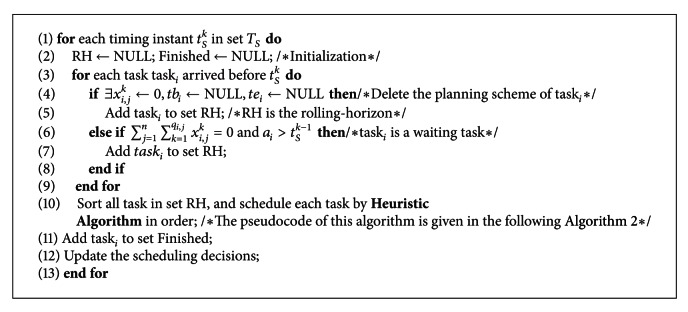
The pseudocode of RHO strategy.

**Algorithm 2:**
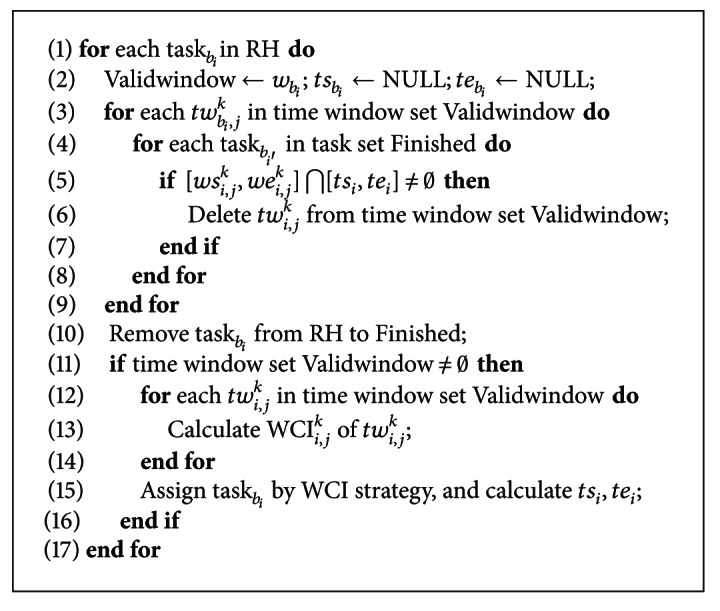
The pseudocode of heuristic algorithm.

**Table 1:** Simulation results in different scenarios.

Problem scale	RH-CIS		RH-DIS		RH-AIS		CIS		DIS		AIS	
	Task benefits	Guarantee ration	Task benefits	Guarantee ration	Task benefits	Guarantee ration	Task benefits	Guarantee ration	Task benefits	Guarantee ration	Task benefits	Guarantee ration
100 × 4	396	0.672	401	0.683	397	0.674	382	0.642	397	0.677	396	0.673
100 × 5	412	0.705	417	0.716	416	0.713	401	0.681	416	0.713	409	0.698
100 × 6	425	0.733	430	0.745	424	0.729	408	0.696	426	0.726	424	0.729
200 × 4	679	0.622	672	0.613	633	0.574	559	0.497	559	0.497	554	0.492
200 × 5	746	0.691	740	0.682	699	0.638	611	0.553	611	0.553	610	0.550
200 × 6	804	0.741	813	0.754	763	0.707	688	0.625	688	0.625	693	0.637
300 × 4	808	0.492	813	0.506	725	0.442	665	0.409	663	0.405	658	0.392
300 × 5	890	0.551	900	0.562	812	0.506	745	0.458	744	0.453	736	0.450
300 × 6	1027	0.649	1021	0.642	933	0.580	873	0.541	877	0.549	860	0.531
400 × 4	897	0.423	895	0.413	801	0.366	729	0.339	722	0.332	717	0.326
400 × 5	992	0.468	990	0.461	876	0.403	815	0.377	802	0.370	800	0.364
400 × 6	1152	0.549	1143	0.541	1025	0.487	959	0.448	953	0.441	946	0.435
